# Assessing the impact of a single qualitative fecal immunochemical test on colonoscopy prioritization and mortality in risk-stratified patients with suspected colorectal cancer: a retrospective cohort study

**DOI:** 10.1016/j.lana.2025.101201

**Published:** 2025-08-11

**Authors:** Felipe F. Quezada-Diaz, Johanna Acevedo, Maite González, Andrea Tello, Richard Castillo, Carlos Morales, Erik Manríquez, Valentina Duran, Felipe Mena, Catherine Le-Bert, Manuel Cabreras, Ángelo Fulle, Gonzalo Carvajal, Pamela Briones, Bruno Nervi, Rodrigo Kusanovich

**Affiliations:** a Servicio de Cirugía, Unidad de Coloproctología, Complejo Asistencial Doctor Sótero Del Río, Santiago, Chile; b Instituto de Ciencias e Innovación en Medicina, Facultad de Medicina Clínica Alemana Universidad del Desarrollo, Santiago, Chile; c Centro para la Prevención y el Control del Cáncer (CECAN), Santiago, Chile; d Escuela de Medicina. Pontificia Universidad Católica de Chile, Santiago, Chile; e Departamento de Hematología y Oncología, Facultad de Medicina, Pontificia Universidad Católica de Chile, Santiago, Chile

**Keywords:** Colorectal neoplasms, Cancer screening, Colonoscopy, Health priorities, Fecal occult blood test

## Abstract

**Background:**

Performing fecal immunochemical tests in symptomatic individuals at low-or moderate risk for suspected colorectal cancer could help prioritize candidates for colonoscopies. The objective of this study was to assess the diagnostic accuracy of the fecal immunochemical test (FIT) in symptomatic individuals at low-or moderate risk of colorectal cancer and explore association with survival.

**Methods:**

We conducted a retrospective cohort study between December 2016 and July 2024 from a single-center, public hospital in Chile. Adults (≥18 years-old) individuals were included, those with symptoms suggestive of colorectal cancer and set for evaluation via colonoscopy. Symptomatic individuals with suspected colorectal cancer were stratified as high risk or low/moderate risk by a trained nurse according to 2015 NICE guidelines. Subsequently, high risk patients were directly referred for colonoscopies, while low/moderate risk patients underwent a single qualitative FIT and prioritized to colonoscopy based on results. Main outcomes were FIT diagnostic accuracy for colorectal cancer, overall mortality, and colorectal cancer-specific mortality.

**Findings:**

A total of 394 out of 1304 participants (30%) were classified as high risk. The remaining 910 (70%) were categorized as low/moderate risk and were referred for FIT. From these, 808 (89%) individuals were tested and had results for FIT. Regarding the diagnostic accuracy of the FIT, sensitivity was 96% and specificity reached 66.8%, with a negative value of 99.8%. Low/moderate risk positive FIT (FIT+) and high-risk participants had higher mortality rates vs. low/moderate risk negative FIT (FIT−) individuals. Time-to-event analysis confirmed a lower cumulative mortality in low/moderate risk FIT− patients. A multivariable Cox regression model showed a consistently lower risk of death in this group, while a non-significant trend towards increased mortality was observed in low/moderate risk FIT+ individuals after 30 months.

**Interpretation:**

In symptomatic individuals at low or moderate risk, a single qualitative FIT was associated with high sensitivity and moderate specificity for colorectal cancer detection. FIT may help prioritize colonoscopy in low-resource settings, but further prospective validation is warranted.

**Funding:**

This research was partially funded by 10.13039/501100020884ANID10.13039/501100018735FONDAP152220002 (CECAN).

**Research in context**

**Evidence before this study**

We searched PubMed, Google Scholar, EMBASE, and MEDLINE for studies published between 2019 and 2025 evaluating the use of fecal immunochemical tests (FIT) to triage symptomatic patients for colonoscopy in the context of suspected colorectal cancer (CRC). Search terms included: “Faecal immunochemical test” AND “Symptomatic” AND “Colon Cancer.” This was supplemented by internet searches (Google), screening of reference lists, and expert input. We identified three key meta-analyses, all focused on quantitative FIT. The first examined its use in patients with iron-deficiency anemia and found high sensitivity for advanced colorectal neoplasia, supporting its role in prioritizing high-risk patients when endoscopic resources are constrained. The second evaluated a single quantitative FIT in symptomatic patients and concluded that low fecal hemoglobin thresholds can reliably exclude CRC, endorsing its use as a triage tool to optimize colonoscopy allocation. The third confirmed that, regardless of CRC prevalence, quantitative FIT demonstrates high sensitivity for CRC detection; however, its negative predictive value is particularly robust in studies restricted to symptomatic populations.

**Added value of this study**

This study provides novel evidence on the use of a single qualitative FIT to triage symptomatic patients awaiting colonoscopy in a resource-limited setting. Unlike quantitative tests, qualitative FITs are more accessible and less expensive. Our findings support their practical implementation as a feasible and effective triage strategy in such contexts.

**Implications of all the available evidence**

Integrating FIT into diagnostic pathways for symptomatic patients may improve efficiency and reduce unnecessary colonoscopies, particularly in settings with constrained endoscopic capacity. A FIT-based triage approach could expedite access for high-risk, FIT-positive individuals while safely deferring investigation in lower-risk, FIT-negative patients. This strategy promotes more selective use of colonoscopy, aiming to increase diagnostic yield by prioritizing individuals more likely to harbor significant pathology.

## Introduction

Worldwide, colorectal cancer accounts for 10% of all cancer cases, posing a growing public health challenge as incidence rates are projected to rise.^1^^,^^2^ Early detection through screening improves prognosis and survival^3^ with the American Cancer Society and the U.S. Preventive Services Task Force recommending routine screening for individuals aged 45 and older at average risk. Screening options include colonoscopy every 10 years, flexible sigmoidoscopy every 5 years, or an annual fecal immunochemical test (FIT), a non-invasive test that detects blood in stool samples.^4^

Colonoscopy remains the gold standard for colorectal cancer diagnosis, allowing for detection and removal of lesions or polyps.^5^ However, colorectal cancer -related symptoms are often nonspecific and have low predictive value,^6^ resulting in numerous unnecessary colonoscopies that offer little clinical benefit. FIT provides a non-invasive approach that can help prioritize symptomatic patients who are most likely to benefit from colonoscopy, optimizing resource allocation and reducing the burden on healthcare systems. This approach is particularly relevant in settings with limited resources or high demand for diagnostic procedures.^7^, ^8^, ^9^

In Chile, colorectal cancer is the second most common cancer with high incidence and mortality rates mirroring global trends.^10^^,^^11^ Since 2013, the General Health Guarantees (GES) program in Chile has mandated a 45-day deadline for diagnostic colonoscopy in individuals with suspected colorectal cancer and ensures treatment and follow-up for diagnosed cases.^12^ However, the program faces several challenges, including an extensive waiting list and encompassing both high- and low-risk individuals. These delays are likely to negatively impact the timeliness of diagnosis and treatment outcomes.

This study aims to evaluate the potential use of a single qualitative FIT as a diagnostic tool in a cohort of symptomatic, risk-stratified patients on a colonoscopy waiting list at a resource-limited hospital, with a focus on its impact on colorectal cancer -diagnosis and long-term overall and colorectal cancer -specific mortality. Our hypothesis is that a single qualitative FIT can identify individuals at higher risk of colorectal cancer from the colonoscopy waiting list, enabling prioritization.

## Methods

This was a retrospective cohort study conducted at a single public hospital in Chile. Participants were prospectively enrolled into the Early Colorectal Cancer Detection Program (DIPRECC) between December 1st, 2016, to December 31st, 2019. No prior sample size calculation was performed, as a complete sample of all eligible patients during the study period was included. Adult individuals (aged ≥ 18 years) with colorectal cancer-related symptoms, referred for colonoscopy by general practitioners and subsequently added to the hospital colonoscopy waiting list, were enrolled in the study. Patients were excluded if they had a history of colorectal cancer, required urgent colonoscopy or intervention due to major lower gastrointestinal bleeding, impending large bowel obstruction/perforation, or failed to provide a FIT sample. Before being added to the waiting list, all participants were evaluated by a trained nurse, who administered a structured questionnaire, collected clinical history, and classified them as high risk or low/moderate risk using as reference the 2015 National Institute for Health and Care Excellence (NICE) guidelines.^13^ Patients were classified as high-risk by trained nursing staff if they presented with rectal bleeding within one month, a palpable abdominal or rectal mass, or imagenological evidence (computerized tomography scan and/or abdominal ultrasound) of colorectal neoplasia. In select cases, nursing staff could also designate patients as high risk based on subjective clinical evaluation when a colonoscopy was deemed clinically beneficial.

All other patients were classified as low/moderate risk and underwent a qualitative FIT (Monlab Test ®, Barcelona, Spain–sensitivity: 98.3%; specificity: 99.6%, as reported by manufacturer). According to the manufacturer’s specifications, a FIT returns a positive result when the sample contains ≥40 μg of hemoglobin per gram of feces.

Low/moderate risk with a positive FIT result (FIT+) were prioritized for colonoscopy, while those with a negative FIT result (FIT−) patients were assigned lower priority, and scheduled colonoscopy according to standard waiting times.

### Variables

Demographic and clinical information of participants was obtained via health surveys conducted by a nursing professional. Symptoms were self-reported by participants; however, anemia was confirmed through laboratory records as hemoglobin levels <12 g/dL in women or <13 g/dL in men, based on tests performed after study inclusion.

Colorectal cancer diagnoses were validated using the Cancer Hospital Registry at Complejo Asistencial Doctor Sótero del Río Hospital, which includes only biopsy- or histopathology-confirmed cases. Due to real-world constraints within the healthcare system, not all participants underwent the reference standard. To minimize verification bias, additional cases of colorectal cancer were identified through the GES notification system, a national mandatory system that ensures timely access to cancer-related services within the Chilean healthcare system. Survival status and cause of death were verified using official death certificates issued by the Chilean Civil Registry and Identification Service.

Patients who did not return a FIT were included in descriptive analyses but excluded from diagnostic accuracy and survival analyses.

### Outcomes

Primary outcomes included colorectal cancer diagnosis, all-cause mortality, and colorectal cancer -specific mortality.

Colorectal cancer diagnosis was confirmed by biopsy or histopathological analysis of malignant neoplasia in the colon or rectum.

All-cause mortality was defined as any death occurring after enrolling into the study, while colorectal cancer -specific mortality was defined as death occurring after the test/enrollment date, with colorectal cancer recorded as the primary or underlying cause of death (ICD-10 codes C18, C19, and C20). Follow-up extended from study enrollment until the date of death, July 21, 2024, or until completing 60 months (5 years) of follow-up–whichever occurred first–ensuring comparable observation periods for all participants.

### Exposure

Exposure was based on risk categorization: High risk or low/moderate risk. The Low/moderate risk group was further subdivided based on FIT results as FIT+ or FIT−.

### Statistical analysis

Demographic and clinical variables, stratified by colorectal cancer risk were analyzed by descriptive statistics. Categorical variables were compared using the chi-squared (χ^2^) test or Fisher’s exact test, as appropriate, while non-parametric continuous variables were compared using the Kruskal–Wallis test. The diagnostic accuracy of FIT was evaluated by calculating sensitivity, specificity, positive predictive value (PPV), negative predictive value (NPV), positive likelihood ratio (LR+), and negative likelihood ratio (LR−).

Cumulative mortality curves were estimated using 1 − survival probabilities (1 − S(t)) from the Kaplan–Meier method and compared using the log-rank test. The group without FIT was included in baseline descriptive analyses but excluded from mortality analyses, as it lacked a comparable risk classification. No patients were lost to follow-up, as survival status was systematically ascertained through national mortality records.

To estimate the relative risk of all-cause mortality, a multivariable Cox regression model was fitted including patients with both positive and negative FIT results, adjusting for age (as a continuous variable), sex assigned at birth, and a time-dependent interaction term for the FIT+ subgroup (split at 30 months). The high-risk group was used as the reference category for all survival models. A time-dependent interaction term was included only for the FIT+ subgroup, as no violation of proportional hazards was detected for the FIT-subgroup (p = 0.32).

**Supplementary Fig. S1:**
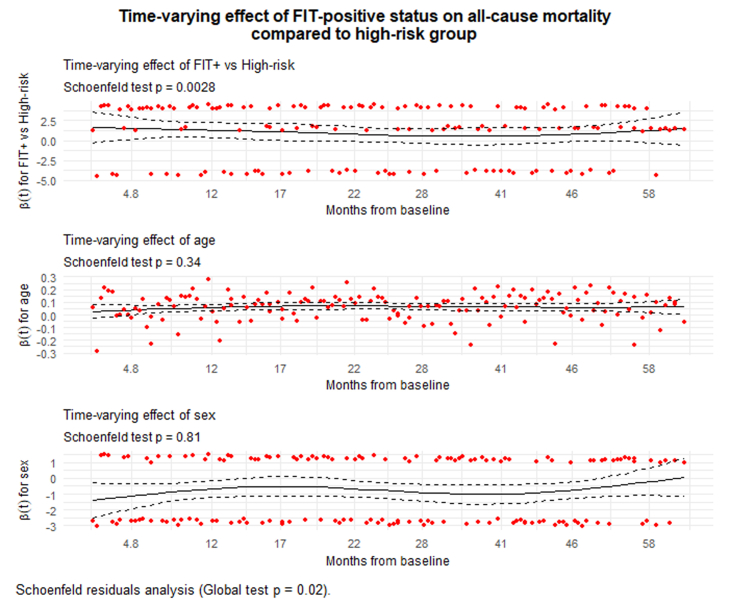


The choice of the 30-month time threshold was based on a visual inspection of the Schoenfeld residuals, which showed a violation of the proportional hazards assumption for the FIT+ subgroup (p = 0.0028), with an upward trend in the effect starting from that point (Supplementary Fig. S1), as well as on a sensitivity analysis aimed at identifying the optimal temporal cut-off point (Supplementary Table S1). The model incorporating an interaction at 30 months showed the best balance between statistical significance (p = 0.045), while other tested cut-off points (between 12 and 36 months) did not reach statistical significance. For time-to-event analyses (including cumulative incidence and Cox regression models), follow-up time was truncated at 60 months to ensure comparability across groups. Patients without events were censored at the 60-month mark or at the end of follow-up, whichever occurred first.

All statistical analyses were conducted in R (version 4.3.2), using a two-sided significance level of 5% and 95% confidence intervals.

### Ethical approval

This observational study posed no risk to participants. All patients received medical care in accordance with the timelines and protocols established by the GES program. Data were anonymized, and the study was conducted in compliance with the Declaration of Helsinki and all applicable national and institutional regulations. Ethical approval was obtained from the Scientific Ethics Committee for Health Sciences at Complejo Asistencial Dr. Sótero del Río, which also granted a waiver of informed consent for the use of data as part of a clinical protocol implementation.

### Role of the funding source

The funding source had no role in the study design; in the collection, analysis, or interpretation of data; in the writing of the report; or in the decision to submit the manuscript for publication.

## Results

**Fig. 1:**
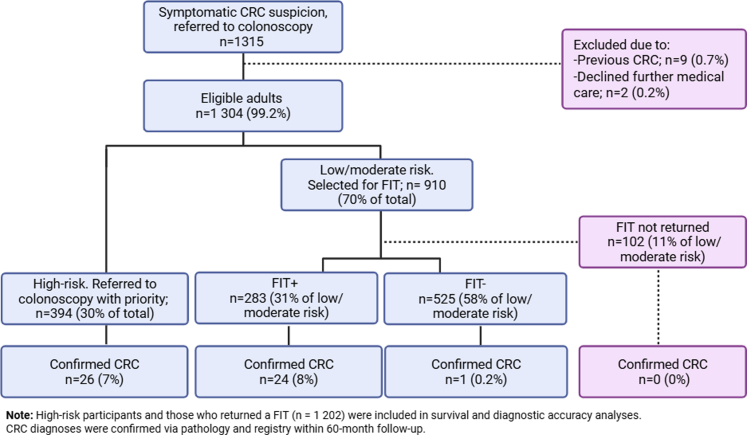
Study Flowchart. CRC = Colorectal Cancer; FIT = Fecal immunochemical test; FIT+ = Positive FIT; FIT− = Negative FIT.

A total of 1315 symptomatic patients with colorectal cancer suspicion were initially enrolled in the study. Of these, 9 (0.7%) were excluded due to previous colorectal cancer diagnosis after a second assessment and 2 were excluded after explicitly declining medical care. Among the remaining 1304 patients, 394 were classified as high-risk (30%) and prioritized for colonoscopy, while 910 (70%) were classified as low/moderate risk and referred for FIT. A total of 808 patients (89%) completed FIT, while 102 (11%) did not return the sample and were therefore excluded from the final analysis (Fig. 1). The low/moderate risk was further divided into FIT+ (n = 283), and FIT− (n = 525).

Baseline clinical characteristics and outcomes are summarized in Table 1. High-risk patients had a higher proportion of prior colonoscopy and clinical follow up for polyps. They also experienced shorter waiting times for colonoscopy, consistent with the established prioritization protocol. However, there were significant differences in the overall colonoscopy completion rate across groups, with the highest proportion observed among low/moderate risk FIT+ (88%) compared to high-risk patients, low/moderate risk FIT−, and those who did not return the FIT.

**Table 1:** Baseline characteristics stratified by colorectal cancer risk.

Variable	Total (n = 1304)	High-Risk (n = 394)	Low/Moderate Risk (n = 910)			p-value^c^
			FIT+ (n = 283)	FIT− (n = 525)	FIT not returned (n = 102)	
Follow-up (months)^a^	60 [0.2–60]	60 [1.9–60]	60 [0.2–60]	60 [1.7–60]	60 [0.2–60]	0.0056
Age (years)	62 [18–92]	63 [18–92]	63 [35–85]	62 [24–90]	59 [33–87]	0.12
**Sex n (%)**						
Male	408 (31)	136 (35)	93 (33)	145 (28)	34 (33)	0.13
Female	896 (69)	258 (65)	190 (67)	380 (72)	68 (67)	
**Symptoms, n (%)**						
Weight loss	380 (30)	111 (29)	95 (34)	156 (30)	18 (18)	0.044
Missing data	17 (1.3)	13 (3.3)	0 (0)	0 (0)	4 (3.9)	
Diarrhea	247 (19)	71 (19)	66 (23)	97 (18)	13 (13)	0.14
Missing data	16 (1.2)	11 (2.8)	0 (0)	0 (0)	5 (4.9)	
Constipation	473 (37)	134 (35)	98 (35)	202 (38)	39 (40)	0.56
Missing data	16 (1.2)	12 (3)	0 (0)	0 (0)	4 (3.9)	
Rectal bleeding^b^	544 (42)	164 (43)	154 (54)	189 (36)	37 (37)	<0.0001
Missing data	12 (0.9)	9 (2.3)	0 (0)	0 (0)	3 (2.9)	
Anemia diagnosed during prioritization	160 (12)	59 (15)	43 (15)	48 (9)	10 (10)	0.017
Missing data	7 (0.5)	3 (0.8)	0 (0)	0 (0)	4 (3.9)	
Previous COL	290 (23)	123 (32)	56 (20)	96 (18)	15 (15)	<0.0001
Missing data	21 (1.6)	5 (1.3)	7 (2.5)	4 (0.8)	3 (2.9)	
Family history (any)	172 (13)	44 (11)	47 (17)	67 (13)	14 (14)	0.19
Missing data	25 (1.9)	5 (1.3)	8 (2.8)	8 (1.5)	4 (3.9)	
1st degree relative	126 (10)	28 (7)	33 (12)	55 (10)	10 (10)	0.20
Missing data	0 (0)	0 (0)	0 (0)	0 (0)	0 (0)	
<50 yr-old relative	24 (2)	4 (1)	5 (2)	13 (2)	2 (2)	0.43
Missing data	0 (0)	0 (0)	0 (0)	0 (0)	0 (0)	
**Surveillance, n (%)**						
Polyps	71 (6)	33 (9)	15 (5)	22 (4)	1 (1)	0.0074
Missing data	28 (2.1)	7 (1.8)	9 (3.2)	8 (1.5)	4 (3.9)	
Inflammatory bowel disease	5 (0)	1 (0)	1 (0)	3 (1)	0 (0.0)	0.90
Missing data	36 (2.8)	9 (2.3)	9 (3.2)	14 (2.7)	4 (3.9)	
**Post-evaluation procedures, n (%)**						
Colonoscopy performed	739 (57)	261 (66)	248 (88)	229 (44)	1 (1)	<0.0001
Missing data	0 (0)	0 (0)	0 (0)	0 (0)	0 (0)	
Time colonoscopy (days)	56 [2–1668]	37 [2–1423]	48 [8–1668]	137 [7–1526]	15 [15–15] ∗∗	<0.0001
**Lifestyle habits, n (%)**						
Smoking	332 (26)	96 (25)	72 (26)	124 (24)	40 (40)	0.0062
Missing data	16 (1.2)	4 (1)	4 (1.4)	5 (1)	3 (2.9)	
Alcohol consumption	279 (22)	92 (24)	71 (25)	98 (19)	18 (18)	0.10
Missing data	9 (0.7)	4 (1)	1 (0.4)	0 (0)	4 (3.9)	

FIT = Fecal immunochemical test; FIT+ = Positive FIT; FIT− = Negative FIT.

a Follow-up truncated at 60 months.

b High-risk: bleeding ≥1 month; Low/Moderate-risk: bleeding >1 month.

c p-values for categorical variables calculated using χ^2^ test (expected cell counts ≥5) or Fisher’s exact test (cell counts <5); continuous/non-parametric variables analyzed with Kruskal–Wallis test. Missing data handled by exclusion per analysis.

Regarding presenting symptoms, both the high-risk group and low/moderate risk FIT+ subgroup had a higher proportion of anemia compared to other groups. Additionally, rectal bleeding was most frequently reported in the FIT+ subgroup, which in this group corresponded to bleeding for more than one month, in accordance with triage criteria.

A total of 51 participants were diagnosed with colorectal cancer. The proportion of confirmed colorectal cancer was significantly higher in the low/moderate risk FIT+ subgroup (8%) vs. high-risk group or low/moderate risk FIT-subgroup (p < 0.0001). A total of 161 deaths (12%) were recorded during the study. An associated significantly higher mortality was noted in the affected high risk and low/moderate risk FIT+ patients. In contrast, the FIT− subgroup exhibited the lowest all-cause mortality and had no colorectal cancer -specific deaths during the 60-month follow-up period (Table 2). Other variables such as median age or BMI did not show significant differences.

**Table 2:** Diagnostic accuracy of qualitative fecal immunochemical test.

Variable	Total (n = 1304)	High-Risk (n = 394)	Low/Moderate Risk (n = 910)			p-value^a^
			FIT+ (n = 283)	FIT− (n = 525)	FIT not returned (n = 102)	
Confirmed CRC diagnosis, n (%)	51 (4)	26 (7)	24 (8)	1 (0.2)	0 (0)	<0.0001
**All-cause mortality, n (%)**						
Full follow-up, n (%)	161 (12)	65 (16)	42 (15)	45 (9)	9 (9)	0.0011
≤30 months, n (%)	93 (7)	45 (11)	17 (6)	28 (5)	3 (3)	0.0093
**Cause of death (n/% of deaths)**^b^						
CRC-specific	14/161 (9)	6/65 (9)	7/42 (17)	1/45 (2)	0/9 (0)	0.11
Other	147/161 (91)	59/65 (91)	35/42 (83)	44/45 (98)	9/9 (100)	

FIT = Fecal immunochemical test; FIT+ = Positive FIT; FIT− = Negative FIT.

a Chi-squared (χ^2^) test was used when expected cell counts were ≥5; Fisher’s exact test was used for sparse data (expected counts <5).

b Percentages calculated relative to total deaths per group. Missing data: No patients lost to follow-up.

Importantly, we evaluated the diagnostic accuracy of the qualitative FIT for the low/moderate risk group. As shown in Table 3, 24 out of 25 confirmed colorectal cancer cases were correctly identified by the FIT (True positives), yielding a high sensitivity of 96%. Conversely, the analysis identified 524 true negatives and 259 false positives, which results in a specificity of 66.8%. Full results are presented in Table 3.

**Table 3:** Diagnostic accuracy of fecal immunochemical test (FIT) for colorectal cancer detection.

	Patients referred to FIT n = 808
**Colorectal cancer n**	25
True positive	24
False negative	1
False positive	259
True negative	524
Sensitivity % (95% CI)	96.0 (76.6–99.9)
Specificity % (95% CI)	66.9 (63.5–70.2)
PPV % (95% CI)	8.5 (5.5–12.3)
NPV % (95% CI)	99.8 (98.9–100)
LR+	2.90
LR−	0.06

FIT: Fecal immunochemical test; CI: confidence interval.

95% confidence intervals (CI) calculated using Clopper-Pearson exact method for proportions.

**Fig. 2:**
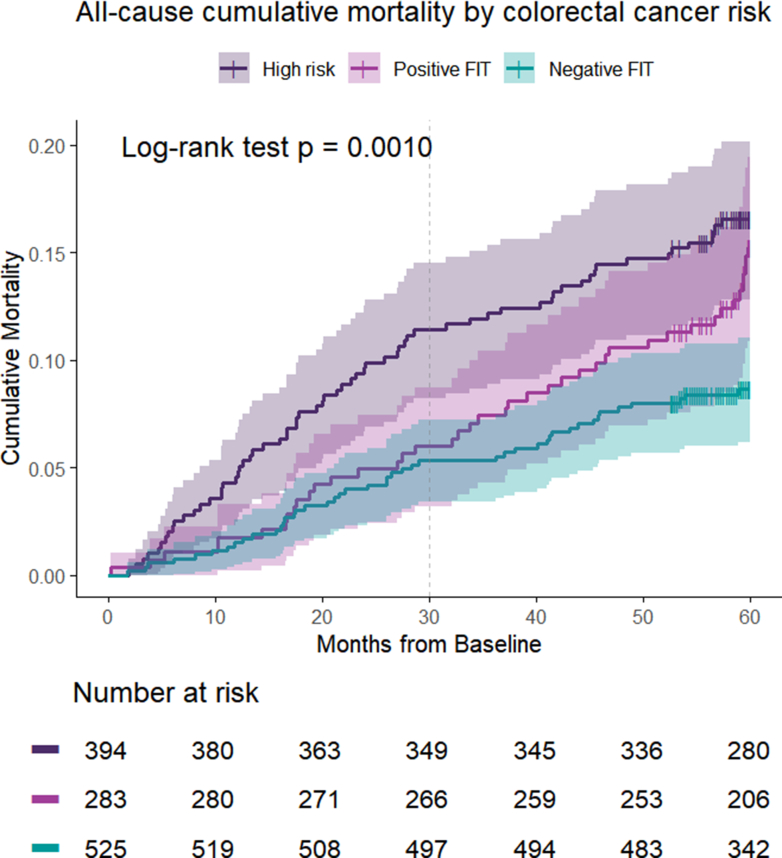
All-cause Cumulative Mortality by Colorectal Cancer Risk. FIT = Fecal immunochemical test.

Mortality differences between groups were initially assessed using cumulative mortality curves (Fig. 2), which revealed higher mortality probabilities for patients in the high-risk group and those with a FIT+, compared to the FIT-subgroup (log-rank test, p < 0.0001). While this method does not account for time-varying effects, it highlights overall differences in mortality risk across strata.

In the multivariable Cox analysis adjusted for age, sex, and time (including a time-dependent interaction term exclusively for the FIT+ subgroup; see Methods), the FIT-group showed a 50% lower risk of all-cause mortality compared to the high-risk group (HR: 0.50; 95% CI: 0.34–0.74; p < 0.001). Among FIT+ individuals, a biphasic time pattern was identified: During the first 30 months, there was a 51% reduction in mortality risk (HR: 0.49; 95% CI: 0.28–0.85; p = 0.011). After 30 months, a non-significant trend toward increased mortality was observed (HR: 1.40; 95% CI: 0.83–2.35; p = 0.21), equivalent to a 40% higher risk compared to the initial period (Table 4).

**Table 4:** Multivariable Cox model for all-cause mortality by CRC risk classification, with time-dependent analysis.

Risk factor	HR (IC del 95%)	p-value
Negative FIT	0.50 (0.34–0.74)	0.0004
Positive FIT (0–30 months)	0.49 (0.28–0.85)	0.011
Positive FIT (>30 months)^a^	1.40 (0.83–2.35)	0.21
Age (per year)	1.06 (1.05–1.08)	<0.0001
Male sex (ref: Female)	2.11 (1.37–2.90)	<0.0001
**Interaction: Positive FIT × >30 months**	2.85 (1.37–5.93)	0.0050

FIT = Fecal immunochemical test.

Reference category: High-risk patients; female sex.

Time was split at 30 months to evaluate changes in the effect of FIT-based stratification.

Schoenfeld global test: χ2 = 3.96 (df = 5), p = 0.56.

Model adjusted for age and sex.

a HR > 30 months = HR (Positive FIT) × HR (Interaction).

This change in risk was confirmed by a statistically significant time-dependent interaction term (HR: 2.85; 95% CI: 1.37–5.93; p = 0.0050), suggesting a time-varying effect specific to the FIT+ subgroup.

## Discussion

This study showed that a single qualitative FIT was associated with a high sensitivity (96%) and moderate specificity (66.8%) for colorectal cancer detection in a cohort of symptomatic patients referred for colonoscopy. Notably, symptomatic FIT-subgroup patients displayed lower all-cause mortality and only 1 patient with colorectal-specific death when compared to both high risk group and FIT+ subgroup.

Our findings align with the current literature. The implementation of a qualitative FIT demonstrated high sensitivity but moderate specificity due to a high proportion of false positives. A multicenter study of over 9800 patients showed that using a 2 μg/g hemoglobin (Hb)/feces cut-off maximized sensitivity at 97.0% but reduced specificity to 64.9%.^14^ Importantly, the study concluded that FIT-at this cut-off may effectively rule out colorectal cancer, while FIT+ identifies patients requiring urgent investigation. A study in Southwestern England reported lower FIT sensitivity (84.3%) but higher specificity (85.0%) in low-colorectal cancer -risk patients.^15^ Predictive values from this study were consistent with our findings, with PPV: 7.0% vs. 8.5% and NPV: 99.8% vs. 99.8% (see Table 3). The reduced specificity of our qualitative FIT may stem from factors such as a smaller sample size (n = 808) and comorbidities contributing to incidental bleeding.^16^ As colorectal cancer symptoms are often nonspecific, this increases false positives and lowers specificity. Nonetheless, our qualitative FIT showed sensitivity and specificity levels comparable to quantitative FITs with low cut-offs (∼2 μg/g). Notably, risk stratification in our study followed NICE guidelines, which recommend FITs for adults with suspected colorectal cancer, particularly those with rectal bleeding or anemia.^13^ We did not exclude patients with active rectal bleeding or iron-deficiency anemia from our analysis, even though these conditions could contribute to false-positive results.

Recent studies confirm that patients with high-risk symptoms, including those in this subset, can benefit from FITs.^17^, ^18^, ^19^, ^20^, ^21^ In our study, overall mortality was elevated across all groups, with particularly high mortality among FIT-positive individuals (adjusted for age and sex assigned at birth), suggesting that a positive FIT may also serve as a marker of frailty. This mortality trend is likely driven by the characteristics of our patient population—a public tertiary care center that primarily manages high-risk patients with significant comorbidities and socioeconomic challenges, such as low income, which may collectively contribute to the high death rates observed. Interestingly, the risk of death among FIT-positive patients was lower during the first 30 months of follow-up, possibly reflecting the clinical benefit of expedited colonoscopy, which was achieved in 88% of this group. Additionally, a large study of over 400 000 healthy individuals found that fecal Hb levels as low as 4 μg/g were associated with both colorectal cancer -specific and all-cause mortality.^21^ These findings support incorporating FIT into routine screenings, even without colorectal cancer suspicion, as a strategy to improve overall survival in the general population. A South Korean study of the National Cancer Screening program showed that FIT+ was associated with higher all-cause, and colorectal cancer -specific mortality compared to FIT-individuals.^22^ Another retrospective study demonstrated that FIT+ individuals that do not comply with colonoscopy double their risk of colorectal cancer -specific mortality.^23^ Recently, a Swedish study demonstrated that routine FIT in the general population effectively reduces colorectal cancer mortality^24^: In 2022, the Association of Coloproctology of Great Britain and Ireland, in collaboration with the British Society of Gastroenterology, developed a guideline for the use of FIT in individuals with suspected colorectal cancer.^25^ Interestingly, a trained nurse identified a higher proportion of younger individuals (<50 years old) as high-risk. These patients were also more likely to have undergone previous colonoscopies and be under surveillance for polyps. This trend also aligns with recent studies showing a higher incidence of colorectal cancer in younger patients, who are often diagnosed at more advanced stages with poorer prognoses.^26^ Consistent with previous studies, FIT+ subgroup had a higher incidence of rectal bleeding (not reported within 1 month),^27^ anemia as found after the enrollment,^28^ and exhibited higher mortality rates compared to FIT-subgroup.^29^

This study has both strengths and limitations. One key strength is the use of qualitative FITs, which provide a cost-effective alternative in resource-limited settings. This simplicity represents a significant advantage in healthcare settings such as Latin America, as it does not require specialized equipment and avoids the higher costs associated with quantitative determinations. Furthermore, using a qualitative test simplifies its integration across multiple centers, as these tests do not require the establishment of specific cut-off values for analysis. This study is limited by its retrospective design and its applicability to a Chilean population, which may reduce the generalizability of the findings to other ethnic or geographic groups.

Conversely, colonoscopy referrals and prioritizations were generated by training nursing staff based on different criteria, without a standardized protocol, but following the high-risk criteria previously mentioned. Although this can certainly lead to bias, it also gives the study a pragmatic character that allows interpretation of its results in a context where staff rotation and variability in criteria among professionals are part of daily practice. Based on our findings—and consistent with international literature—we advocate extending FIT screening to both high-risk and low/mild-risk patients. However, owing to the pragmatic design of our study and limited healthcare resources, we were unable to defer colonoscopy in high-risk patients or implement FIT as a parallel prioritization strategy.

We were also unable to perform colonoscopies on all patients to correlate these results with FIT outcomes. Since the study was conducted during the COVID-19 pandemic, the number of colonoscopies during the pandemic was significantly limited, with delayed screenings, diagnoses, staging, treatment, and follow-up of patients. Another potential limitation is the uneven waitlist time for colonoscopy—particularly in the FIT-subgroup—which may introduce bias due to longer exposure periods. Although we could not fully eliminate this issue, we truncated our survival analyses at 30 and 60 months to minimize follow-up discrepancies. Finally, it is noteworthy that almost 2/3 of low/moderate-risk of patients in our cohort were FIT−; this opens the possibility for improvement in our test. Looking ahead—and pending further validation—our strategy could help reduce unnecessary colonoscopies in certain individuals.

This, in turn, may streamline colonoscopy scheduling, shorten wait times, and ultimately contribute to lower colorectal cancer mortality.

### Conclusion

A single qualitative FIT demonstrated high sensitivity and moderate specificity for colorectal cancer diagnosis in low/moderate-risk individuals. Additionally, a FIT-result was associated with lower all-cause and colorectal cancer -specific mortality. Pending further validation from prospective studies, our findings suggest that using a qualitative FIT in symptomatic low/moderate-risk individuals with suspected colorectal cancer could aid in risk stratification and prioritization for colonoscopy. Low/moderate-risk individuals with a FIT+ result may benefit from expedited colonoscopy, while those with a negative result—given their association with lower mortality—could be assigned a lower priority. This approach may help reduce colorectal cancer -specific mortality while optimizing colonoscopy resources, particularly in healthcare systems with limited capacity.

In countries lacking the infrastructure or medical specialists to implement widespread colorectal cancer screening programs, stratifying colonoscopy waiting lists using fecal occult blood tests for individuals with suspected colorectal cancer symptoms may help lower both all-cause and colorectal cancer -specific mortality. These findings underscore the need for a prospective study to validate the predictive value of FIT before colonoscopy in suspected colorectal cancer cases, followed by an interventional study to assess the impact of prioritizing FIT+ patients.

## Contributors

FQ: conceptualization, data curation, formal analysis, funding acquisition, investigation, methodology, project administration, resources, software, supervision, validation, visualization, writing—original draft, and writing—review & editing.

JA: formal analysis, funding acquisition, investigation, methodology, project administration, resources, software, supervision, validation, visualisation, writing—original draft, and writing—review & editing.

MG: conceptualization, data curation, formal analysis, investigation, methodology, resources, software, validation, visualization, writing—original draft, and writing—review & editing.

AT: data curation, formal analysis, investigation, methodology, project administration, supervision, validation, visualization, writing—original draft, and writing—review & editing.

RC: supervision, validation, visualization, writing—original draft, and writing—review & editing.

CM: data curation, investigation, methodology, validation, visualization, writing—original draft, and writing—review & editing.

EM: data curation, investigation, methodology, validation, visualization, writing—original draft, and writing—review & editing.

VD: methodology, validation, visualization, writing—original draft, and writing—review & editing.

FM: validation, visualization, writing—original draft, and writing—review & editing.

CL: data curation, validation, visualization, writing—original draft, and writing—review & editing.

MC: validation, visualization, writing—original draft, and writing—review & editing.

AF: validation, visualization, writing—original draft, and writing—review & editing.

GC: validation, visualization, writing—original draft, and writing—review & editing.

PB: validation, visualization, writing—original draft, and writing—review & editing.

BN: funding, validation, visualization, writing—original draft, and writing—review & editing. RK: validation, visualization, writing—original draft, and writing—review & editing.

FQ, MG y AT directly accessed and verified the data reported in the manuscript. FQ, JA and MG are responsible for the decision to submit the manuscript.

## Data sharing statement

De-identified individual participant data supporting the findings of this study, including text, tables, figures, and appendices, will be available upon request. Additional materials such as the study protocol, statistical analysis plan, and analytic code can also be accessed. Data will be shared beginning three months after publication and will remain available for five years. Researchers interested in using the data must submit a methodologically sound proposal outlining their intended use, ensuring alignment with the study’s approved objectives. Requests should be directed to ffquezad@gmail.com, and access will be granted upon signing a data access agreement. The data will be hosted on a third-party platform for the specified period (link to be provided).

## Use of artificial intelligence

During the preparation of this work, the principal author, Felipe Quezada-Diaz, MD, used ChatGPT for grammar and spelling checks. After utilizing this tool, the authors reviewed and edited the content as needed and take full responsibility for the final publication.

## Declaration of interests

All authors have no conflicts of interest.

## Appendix A Supplementary data

Supplemental Data Translated Abstract
