# Supplementary material for: Assessing the impact of a single qualitative fecal immunochemical test on colonoscopy prioritization and mortality in risk-stratified patients with suspected colorectal cancer: a retrospective cohort study
Source: Lancet Reg Health Am. 2025 Aug 11;50:101201. doi: 10.1016/j.lana.2025.101201 (PMC12359272; doi:10.1016/j.lana.2025.101201)
Supplement: Supplemental Data Translated Abstract [file mmc1.docx]

**Editorial disclaimer**

The translation of the Summary was submitted by the authors, and we reproduce it as supplied. It has not been peer reviewed. Our editorial processes have only been applied to the original version in English, which should serve as a reference for this manuscript.

**Resumen**

**Antecedentes:** Realizar pruebas inmunoquímicas fecales (FIT, por sus siglas en inglés) en personas sintomáticas con riesgo bajo o moderado de cáncer colorrectal podría ayudar a priorizar a los candidatos para colonoscopía. El objetivo de este estudio fue evaluar la precisión diagnóstica de la FIT en individuos sintomáticos con riesgo bajo o moderado de cáncer colorrectal y explorar su asociación con la supervivencia.

**Métodos:** Se llevó a cabo un estudio de cohorte retrospectivo entre diciembre de 2016 y julio de 2024 en un hospital público de un solo centro en Chile. Se incluyeron adultos (≥18 años) con síntomas sugerentes de cáncer colorrectal con indicación de evaluación mediante colonoscopía. Los individuos sintomáticos con sospecha de cáncer colorrectal fueron clasificados como de alto riesgo o de riesgo bajo/moderado por una enfermera capacitada, según las guías NICE de 2015. Posteriormente, los pacientes de alto riesgo fueron derivados directamente a colonoscopía, mientras que a los de riesgo bajo/moderado se realizo una FIT cualitativa única y fueron priorizados para colonoscopía según sus resultados. Los resultados principales fueron la precisión diagnóstica de la FIT para cáncer colorrectal, la mortalidad global y la mortalidad específica por cáncer colorrectal.

**Resultados:** Un total de 394 de 1.304 participantes, (30%) fueron clasificados como de alto riesgo. Los 910 restantes (70%) fueron categorizados como de riesgo bajo/moderado y derivados a FIT. De estos, 808 (89%) se sometieron a la prueba y contaban con resultados disponibles. En cuanto a la precisión diagnóstica de la FIT, la sensibilidad fue de 96% y la especificidad alcanzó un 66,8%, con un valor predictivo negativo del 99,8%. Los participantes de riesgo bajo/moderado con FIT positiva (FIT+) y los de alto riesgo presentaron tasas de mortalidad más altas en comparación con los individuos de riesgo bajo/moderado con FIT negativa (FIT−). El análisis de tiempo hasta evento confirmó una menor mortalidad acumulada en los pacientes de riesgo bajo/moderado con FIT−. Un modelo de regresión de Cox multivariado mostró un riesgo consistentemente menor de muerte en este grupo, mientras que se observó una tendencia no significativa hacia una mayor mortalidad en los individuos FIT+ de riesgo bajo/moderado después de 30 meses.

**Interpretación:** En individuos sintomáticos con riesgo bajo o moderado, una única FIT cualitativa se asoció con alta sensibilidad y especificidad moderada para la detección de cáncer colorrectal. La FIT podría ayudar a priorizar la colonoscopía en contextos con recursos limitados, aunque se requiere validación prospectiva adicional.

**Financiamiento:** Esta investigación fue financiada parcialmente por ANID FONDAP 152220002 (CECAN).
