# Supplementary material for: Assessing the impact of a single qualitative fecal immunochemical test on colonoscopy prioritization and mortality in risk-stratified patients with suspected colorectal cancer: a retrospective cohort study
Source: Lancet Reg Health Am. 2025 Aug 11;50:101201. doi: 10.1016/j.lana.2025.101201 (PMC12359272; doi:10.1016/j.lana.2025.101201)
Supplement: Supplementary Table S1 [file mmc2.docx]

**Supplementary Table 1**. Hazard ratios (HR) for all-cause mortality comparing Positive FIT vs High-risk group across different follow-up time split points (Cox model with time interaction, adjusted for age and sex)

| \| Time split (months) \| HR (95% CI) \| p-value \|  \|  \|  \| \| --- \| --- \| --- \| --- \| --- \| --- \| \| 12 \| 0·63 (0·19–2·10) \| 0·45 \|  \|  \|  \| \| 18 \| 0·57 (0·22–1·45) \| 0·24 \|  \|  \|  \| \| 24 \| 0·58 (0·24–1·37) \| 0·21 \|  \|  \|  \| \| **30** \| **0·42 (0·18–0·98)** \| **0·045** \|  \|  \|  \| \| 36 \| 0·61 (0·26–1·43) \| 0·26 \|  \|  \|  \| \| Abbreviations: HR: hazard ratio; CI: confidence interval. \| \|  \|  \|  \|  \| |
| --- | --- | --- | --- | --- | --- | --- | --- | --- | --- | --- | --- | --- | --- | --- | --- | --- | --- | --- | --- | --- | --- | --- | --- | --- | --- | --- | --- | --- | --- | --- | --- | --- | --- | --- | --- | --- | --- | --- | --- | --- | --- | --- |
